# Advances in Synthetic-Biology-Based Whole-Cell Biosensors: Principles, Genetic Modules, and Applications in Food Safety

**DOI:** 10.3390/ijms24097989

**Published:** 2023-04-28

**Authors:** Shijing Chen, Xiaolin Chen, Hongfei Su, Mingzhang Guo, Huilin Liu

**Affiliations:** School of Food and Health, Beijing Technology and Business University (BTBU), Beijing 100048, China; chenshijing@st.btbu.edu.cn (S.C.);

**Keywords:** synthetic biology, whole-cell biosensor, food safety, rapid detection

## Abstract

A whole-cell biosensor based on synthetic biology provides a promising new method for the on-site detection of food contaminants. The basic components of whole-cell biosensors include the sensing elements, such as transcription factors and riboswitches, and reporting elements, such as fluorescence, gas, etc. The sensing and reporting elements are coupled through gene expression regulation to form a simple gene circuit for the detection of target substances. Additionally, a more complex gene circuit can involve other functional elements or modules such as signal amplification, multiple detection, and delay reporting. With the help of synthetic biology, whole-cell biosensors are becoming more versatile and integrated, that is, integrating pre-detection sample processing, detection processes, and post-detection signal calculation and storage processes into cells. Due to the relative stability of the intracellular environment, whole-cell biosensors are highly resistant to interference without the need of complex sample preprocessing. Due to the reproduction of chassis cells, whole-cell biosensors replicate all elements automatically without the need for purification processing. Therefore, whole-cell biosensors are easy to operate and simple to produce. Based on the above advantages, whole-cell biosensors are more suitable for on-site detection than other rapid detection methods. Whole-cell biosensors have been applied in various forms such as test strips and kits, with the latest reported forms being wearable devices such as masks, hand rings, and clothing. This paper examines the composition, construction methods, and types of the fundamental components of synthetic biological whole-cell biosensors. We also introduce the prospect and development trend of whole-cell biosensors in commercial applications.

## 1. Introduction

In recent years, rapid detection technology for ensuring food safety has advanced significantly due to scientists in the field of food safety research. Methods based on the specific recognition of antigens and antibodies, as well as the specific amplification of nucleic acids as sensing elements, have developed fast [1]. The convenience and cost of these methods cannot meet the needs of on-site detection, so large-scale commercial applications remain challenging [2]. On the one hand, simple pretreatment steps cannot guarantee the stability of test results due to the complex composition of the food substrate. On the other hand, the production and purification costs of antibodies, aptamers, nucleic acid amplification enzymes, and primers used by these technologies are high. Especially for high-throughput detection of multiple pollutants, the cost of the detection with the above methods will increase exponentially.

**Figure 1:**
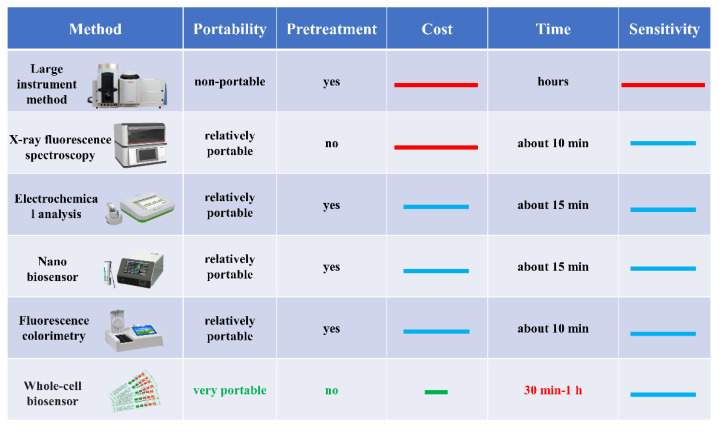
Comparison of different detection methods. The red line in the figure represents “high”, the blue line represents “medium”, and the green line represents “low”.

A synthetic biological whole-cell biosensor is an emerging fast-detection technology for food safety. It uses living cells as a sensing element and converts the information into recognizable signals [3]. The metabolic regulation ability to live cells provides a stable environment for target recognition and signal transformation, allowing them to maintain homeostasis despite external environment changes. Therefore, whole-cell biosensors have strong environmental anti-interference resistance. Whole-cell biosensors are easy to make, inexpensive, and fast to mass-produce because cells can reproduce themselves, allowing all the gene elements within the cell to be amplified automatically through gene replication and cell proliferation. Compared with other detection methods (Figure 1), these advantages make whole-cell biosensors have a promising application prospect in the rapid detection of food safety.

Synthetic biology involves connecting gene fragments based on specific rules so that cells can express signals or products that humans need [4]. With the development of synthetic biology, there have been significant advances in whole-cell biosensors, as synthetic biology has provided whole-cell biosensors with new functional components, genetic modules (logic gate module, memory module, signal amplification module), and assembly theories. Synthetic biology helps the designers of whole-cell biosensors to access more recognition elements targeting food-contaminating molecules, find more convenient forms of signal reporting, and enable smarter detection processes. This article will review the fundamental components, gene circuits, and commercialization application prospects of synthetic biological whole-cell biosensors. Finally, the challenges and development trends of whole-cell biosensors are described.

**Figure 2:**
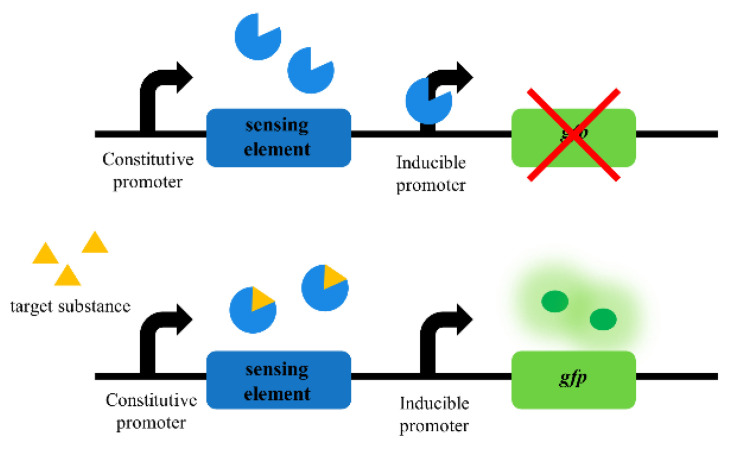
Whole-cell biosensor operating schematic. When the target substance does not exist, green fluorescent protein cannot be expressed. When the target substance exists, green fluorescent protein is expressed.

The basic components of biosensors include the sensing and reporting elements (Figure 2). The sensing element can convert the intensity of a physical or chemical signal to another energy form according to a certain rule, and the reporting element can accept the converted energy and output a signal convenient for human measurement. For example, in the presence of Cu^2+^, the sensing element of a whole-cell biosensor for detecting heavy metals can transmit the signal to the reporting element and then express fluorescence, thus realizing the detection of heavy metals [5]. In whole-cell biosensors, the sensing elements mainly consist of transcription factors and riboswitches, which can transmit detected signals to the reporting elements, causing the cell to express specific proteins that cause visible changes in optical, electrical, and magnetic properties or visual morphology. This element, which can express a specific protein for humans to detect, is called the reporting element.

## 2. Sensing Elements for Whole-Cell Biosensors in Synthetic Biology

The sensing elements of whole-cell biosensors mainly include transcription factors and riboswitches. Transcription factors are a class of protein molecules that can bind to specific sequences upstream of genes and affect gene transcription. Untranslated regions of some mRNA (RNA sequences that do not encode target proteins), known as riboswitches, contain sequences with a certain conformation. These two types of molecules can specifically bind certain chemical substances and change their three-dimensional conformation. After transcription factors undergo a conformational change, their ability to bind the promoter region (A piece of DNA sequence that RNA polymerase recognizes, binds, and begins to transcribe) of genes is changed, thus promoting or inhibiting the transcription process of the gene. For example, MerR is a transcription factor used to detect Hg^2+^. After a riboswitch undergoes a conformational change, its stem ring arrangement changes (The base-paired double-stranded regions on the mRNA form a “stem”, while the unpaired single-stranded regions protrude to form a “ring”), thereby exposing or hiding the ribosome binding sites of mRNA and activating or inhibiting the mRNA translation process. The sensitivity and specificity of the whole-cell biosensor are determined by the ability of the detected substance to bind to the sensing element. Therefore, the key to the successful construction of whole-cell biosensors is to screen the sensing elements that are highly responsive to the detected substances.

### 2.1. Transcription Factor

**Figure 3:**
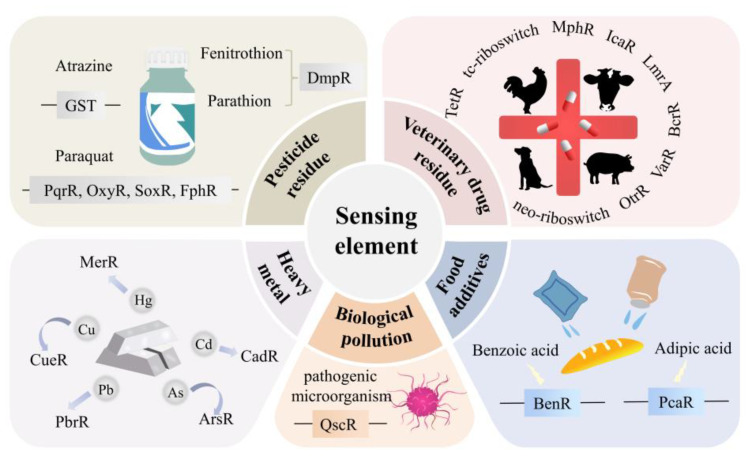
Sensing elements for common target substances in food used in whole-cell biosensors.

When detecting a target substance, the corresponding transcription factor and inducible promoter sequence are found first, and the inducible promoter is used to control the expression of the reporting gene, so as to construct a gene circuit; then, the gene circuit is transferred to chassis cells using plasmid as the carrier. When the target substance is present, chassis cells exhibit the character expressed by the reporting gene. Currently, more than 300 prokaryotic transcription factors have been discovered. Designers of whole-cell biosensors can search a database of prokaryotic transcription factors such as CollecTF1, P2TFA, porTF, and port for transcription factors that identify certain target substances (Figure 3). The traditional transcription factors are mainly discovered and studied by microbiologists. However, the food safety field and the microbiology field focus on different substances, so the known transcription factors discovered by microbiologists cannot meet the needs of food safety detection. Therefore, in the food safety field, most of the hazardous substances to be detected lack corresponding known transcription factors. It is necessary to screen natural organisms for new transcription factors and even construct transcription factors that do not exist. The development of a transcriptomics technique based on high-throughput sequencing has improved the efficiency of screening new transcription factors. For example, Shi et al. [6] added butanol and its analogues propanol and ethanol to yeast cells, identified a promoter that responded specifically to butanol but not to ethanol or propanol, and combined it with the red fluorescent protein gene to construct a whole-cell biosensor for the detection of butanol. When butanol is present, yeast cells will exhibit red fluorescence.

Due to the limited types of transcription factors in nature, the target substances of interest in food safety cannot find the corresponding transcription factors, so it is of great significance to modify or construct transcription factors artificially. Synthetic biology provides strategies for artificially modifying or even constructing transcription factors from scratch for target substances for which natural transcription factors are scarce. Methods commonly used include truncation, chimerism, functional domain mutation, whole-protein mutation, and de novo design. Truncation is to change the performance of a known transcription factor by shortening its length. For example, Tao and others [7] optimized its specificity for cadmium and mercury ions by truncating the transcription factor CadR. In this study, CadR-TC10 and CadR-TC21 were synthesized by removing 10 and 21 amino acids from the C-terminal of CadR, respectively, and used as sensing elements to control green fluorescent protein gene expression and construct an *Escherichia coli* whole-cell biosensor that recognized cadmium and mercury ions but not zinc ions. By combining the target recognition domain of one transcription factor with the gene expression regulation domain of another, chimerism produces a new transcription factor with increased specificity and efficiency in regulating gene expression. For example, Mendoza et al. [8] developed a whole-cell biosensor with good specificity and sensitivity to mercury ions using a chimeric approach. The team replaced the gold ion recognition domain of the transcription factor GolS77 with the mercury ion recognition domain of MerR to construct the GolS* transcription factor. The combination of the GolS* transcription factor and Hg^2+^ can promote the expression of the PgolB promoter-controlled green fluorescent protein gene, thereby converting the whole-cell biosensor for detecting gold ions into a biosensor for detecting mercury ions. Functional domain mutation refers to site-specific mutation of the functional domain (independent functional unit) in the transcription factor. For example, Kasey et al. [9] constructed a saturated mutation library QCMS5 of all five amino acid sites within the recognition domain of the MphR transcription factor, which directly binds macrolides, and screened for a mutant transcription factor with increased specificity and sensitivity for macrolides. The whole-cell biosensor constructed by the mutant transcription factor enables high-throughput directional evolution of the macrolides’ biosynthetic gene pathway. By tuning specificity and sensitivity, the MphR biosensor system may be used in environmental testing, metagenomics, synthetic biology, and metabolic engineering in a way that is completely independent of the macrolides’ biological activity. Whole-protein mutation refers to random mutation of the original transcription factor protein to screen out positive mutants. For example, Chong et al. [10] used error-prone PCR amplification of DmpR genes to introduce random mutations into the whole DmpR protein and screen out transcription factors with improved performance and specific response to organophosphorus, proving that amino acid site mutations outside the functional domain of transcription factors also contribute to an increase in the induced expression level. De novo design means creating transcription factors that do not exist in nature from scratch by utilizing functional domains recognized by enzymes for substrates and antibodies for antigens, and other components. For example, Chang et al. [11] proposed a strategy for designing transcription factors from scratch for new target ligands by fusing single-domain antibodies to monomer DNA binding domains. Dimerization of this transcription factor in the presence of a target ligand activates the DNA-binding function, which then binds to the promoter region of the green fluorescent protein gene, inhibits gene expression, and eventually constructs a transcription-suppressed whole-cell sensor. This strategy has been successful in the construction of caffeine whole-cell biosensors.

Synthetic biology also provides efficient screening schemes for transcription factors engineered or designed from scratch. For example, Liu et al. [12] developed a novel selection circuit based on carbon source utilization that establishes and sustains growth–production coupling over several generations in a medium with maltose as the sole carbon source. Their selection circuit coupled cell fitness to metabolite production with a much lower escape risk, even after multiple rounds of selection, and could also be applied to a small and defined mutational library or a relatively large and undefined one. Thus, the authors identified four new mutations with enhanced L-tryptophan output. This selection circuit provides a new perspective for the optimization of microbial cell factories to produce different metabolites and to discover new genotype–phenotype associations at both single-gene and whole-genome levels. Jia et al. [13] conducted bidirectional screening. Due to the effects on lead ion and activated amp expression, cells with PbrR mutants and strong reactions survived in the presence of ampicillin, whereas weakly bound mutants resulted in cell death during the forward selection process. In the reverse screening phase, PbrR mutant cells with weak zinc binding can survive, because cells that respond to zinc can start the expression of sacB, leading to cell death in the presence of sucrose.

### 2.2. Riboswitch

In 2002, humans discovered the first riboswitch, and currently, there are approximately 20 known types of natural riboswitches [14]. Whole-cell biosensors that use riboswitches as sensing elements are faster in detection compared with transcription factors; this may be because transcription factor signal transformation requires transcription-translation of genes, whereas riboswitch signal transformation only requires translation. There are three main types of riboswitches in prokaryotes. The first type is the adaptive terminator mode. In the absence of target material in the cell, the 5′non-coding structure upstream of the gene forms an anti-terminator structure, allowing RNA polymerase to completely transcribe the gene coding region. However, when the target substance is present in the cell, it binds to the riboswitch, disrupting the stability of the anti-terminator and forming a terminator hairpin structure, which separates the RNA polymerase from the mRNA and halts gene expression. The second type is the adaptation to the Ribosome Binding Site (RBS) model. When there is no target in the cell, the RBS is hidden in the stem ring structure, preventing the ribosome from binding to the mRNA and translation. When the target binds to the riboswitch, the stem ring structure is opened, exposing the ribosome binding site and initiating protein translation. The third type is the adaptive ribozyme pattern. When the target substance binds to the riboswitch region, the self-cleavage activity is activated off the ribozyme, causing mRNA degradation and thus blocking protein translation. Although the theory and mechanism of riboswitches have been well studied, their application in whole-cell biosensors has been limited. In a few whole-cell biosensors, the use of riboswitches as sensing elements has been demonstrated. For example, Wang et al. [15] combined a riboswitch to cobalt/nickel ions with the mCherry gene for the red fluorescent protein to develop highly sensitive and selective cobalt/nickel ion whole-cell biosensors. The sensors were applied to examine the resistance system of Co^2+^/Ni^2+^ in *Escherichia coli* (*E. coli*), and the sensors monitored the effects of genetic deletions. These results indicate that riboswitch-based sensors can be used for the study of related cellular processes. Schneider et al. [16] coupled riboswitches of neomycin and tetracycline, both of which block translation initiation through the same mechanism, and they improved the dynamic range between ON and OFF states of reporter gene expression, enabling the detection of neomycin and tetracycline.

At present, natural riboswitches are unable to meet the needs of hazardous substance detection in the field of food safety research. Target substances that do not have natural riboswitches can be developed by screening, modification, etc. Synthetic biological technology provides a new opportunity for intelligent targeting design of riboswitches. Aptamers are short nucleic acid chains that can bind to target substances with high affinity and specificity, as determined by in vitro screening, and their three-dimensional conformation is closely related to the system environment. Currently, the challenge in this field is to ensure the correct folding of aptamers screened in the in vitro environment in cells. Jang et al. [17] solved this problem using in vitro and in vivo dual screening and developing an artificial caprolactam riboswitch. The team used SELEX (Systematic Evolution of Ligands by Exponential Enrichment) technology to select aptamers from the RNA aptamer library that could specifically bind caprolactam in the buffer environment in vitro. Then, these aptamers were cloned between the promoter and ribosome binding site of the green fluorescent protein gene and transformed into *Escherichia coli* cells. To complete in vivo screening, screening of cells without caprolactam, without fluorescence, and caprolactam with fluorescence was performed. The sensitivity of whole-cell biosensors based on caprolactam riboswitch can reach 50 mM, and can be used to screen industrial strains to improve the yield of caprolactam. The team [18] and Xiu et al. [19] then used this strategy to screen riboswitches that specifically respond to naringin. However, the in vitro and in vivo dual screening strategy has a heavy workload and a low success rate, meaning it cannot fundamentally solve the intracellular application of aptamers problem. A comprehensive examination of the relationship between aptamer sequence, conformation, and environment is the key to fundamentally solving this issue [14,20,21]. In conclusion, there is still a long way to go in the development of artificial riboswitches.

## 3. Reporting Elements for Whole-Cell Biosensors in Synthetic Biology

The reporting element is one of the basic components of a whole-cell biosensor, which can generate signals that can be recognized by humans or instruments. The majority of reporting elements are proteins, while a small number are functional nucleic acids. Their genes enter cells using plasmids as vectors, and their expression is regulated by sensing elements. When selecting a reporting element, it is necessary to comprehensively take into account the detection purpose (qualitative or quantitative), use environment (field or laboratory detection), and sample type of the whole-cell biosensor (solid or liquid, transparent, translucent, or opaque). The various reporting elements that have been developed or are currently being developed can be categorized into eight groups (Table 1): luciferase, fluorescent protein, fluorescent aptamer, pigment-generating enzyme, gas-generating enzyme, magnetosome/magnetic protein, ice nucleation protein, and Curli protein.

### 3.1. Luciferase

Fluorescein can react with oxygen to produce fluorescence, but the rate of this reaction is very slow without enzymatic catalysis. Luciferase can accelerate the reaction when exposed to calcium ions. Therefore, luciferase can be used as a reporting element in whole-cell biosensors. When fluorescence is observed, it indicates the presence of the target substances. Bacterial luciferase (Lux), firefly luciferase (Luc), and aequorin are commonly used as reporting elements. Firstly, the α and β subunits of Lux are encoded by the luxA and luxB genes located in the lux operon, respectively. In addition, the lux operon contains three genes related to the synthesis and recovery of luciferase substrate fatty aldehyde: luxC, luxD, and luxE. Therefore, without the addition of substrate, the expression of all luxABCDE genes in chassis cells can generate fluorescence signals. Secondly, Luc produces visible light by catalyzing the luciferase substrate Benzothiazolyl-Thiazole in the presence of ATP, oxygen molecules, and magnesium ions to produce oxy fluorescein and carbon dioxide. As the reporting element, Luc only needs to be transferred into one gene, reducing the complexity of gene operation. However, since this gene can only catalyze the fluorescence reaction and cannot provide a fluorescein substrate, commercial D-luciferin and other exogenous reaction substrates must be added to the reaction system. Lastly, Aequorin is a calcium–ion binding photoprotein isolated from the bioluminescent Victoria multitube glowing jellyfish. With coelenterazine as the fluorescein substrate, aequorin can covalently bind coelenterazine and oxygen molecules. With the addition of calcium ions, coelenterazine can be oxidized to coelenteramide, producing carbon dioxide and emitting blue light at 460–470 nm. Additionally, aequorin has the advantages of high sensitivity, stability, and no endogenous expression. The luciferase gene is transferred into the plasmid, and its expression is controlled by the inducible promoter, which can be used as a reporting element to realize the visual detection of target substances.

### 3.2. Fluorescent Protein

Fluorescent proteins are the most ideal and also the most widely used whole-cell biosensor reporting element. Presently, fluorescent proteins are used in more than 90% of whole-cell biosensors in synthetic biology. This is because fluorescent proteins are generally expressed by a single gene and can produce stable fluorescence without any substrate. Except for dissolved oxygen, their expression and fluorescence intensity are basically not affected by chassis cell metabolism. Currently, many kinds of fluorescent proteins can be divided into dark blue, blue, cyan, green, yellow, orange, red, and dark red fluorescent proteins and near-infrared fluorescent proteins according to their fluorescence colors. The designers of whole-cell biosensors must consider the following factors when selecting fluorescent proteins [22]. Firstly, chassis cells can effectively express and mature fluorescent proteins, allowing them to generate fluorescent signals with optimal intensity. Secondly, the fluorescent protein should be photostable during the detection process, that is, the fluorescence intensity should remain stable so as to stabilize the detection results. Thirdly, the fluorescent protein should be non-toxic to chassis cells, enabling normal metabolism and expression of chassis cells, and completing testing. Finally, fluorescent proteins should be insensitive to environmental factors in the detection system and should not cause instability in the detection results due to environmental changes. Rathnayake et al. [5] developed a whole-cell bacterial biosensor capable of detecting Cd, Cu, and Zn in soil environments by taking a heavy metal sensitive strain as chassis cell and transferring green fluorescent protein (gfp) into it. When exposed to heavy metals, the whole-cell biosensor can exhibit a rapid and measurable signal response. The results showed that with the increase in heavy metal concentration, the fluorescence signal of gfp gene expression was weaker. The lowest detection limits were determined as 1.42 × 10^−4^, 3.16 × 10^−4^, and 2.42 × 10^−4^ mg/L for Cd, Cu, and Zn, respectively.

### 3.3. Fluorescent Aptamer

RNA aptamer fluorescence reporting element refers to an aptamer capable of forming a strong fluorescence complex upon binding with a non-fluorescent or weakly fluorescent target substance. RNA aptamer is a kind of reporting element. Like fluorescent proteins, it indicates the presence of a target substance with fluorescent signals. The difference between RNA aptamers and fluorescent proteins is that the output of RNA aptamers’ fluorescent signals only require transcription, not translation. Therefore, theoretically, RNA aptamers have shorter output times, but not always. Since each amino acid of a protein element corresponds to three DNA bases and each ribonucleic acid of an RNA element corresponds to one DNA base, the corresponding gene of an RNA element is usually shorter. In component design, RNA is more convenient than protein in cutting, splitting, and splicing, among other actions. Therefore, RNA aptamers have unique advantages as reporting elements. Several aptamers including malachite green aptamer [23], spinach aptamer [24], mango aptamer [25], and broccoli aptamer, are available as reporting elements [26].

### 3.4. Microbial Pigment Reporting Element

Microbial pigment is a class of secondary metabolites produced by animals, plants, and microorganisms. Due to the special chemical pigment structure, which can result in reflection, interference, scattering, and light absorption, the pigment appears in different colors. Pigment synthesis can be controlled by transferring chromogenic enzyme genes into chassis cells using plasmid as carrier. In chassis cells, the whole-cell biosensor is constructed of microbial pigment reporting elements and sensing elements, which can realize the visual detection of target substances. Output signals are produced by microbial pigment reporting elements visible to human eyes, but the fluorescence needs to be observed under ultraviolet light. Therefore, whole-cell biosensors composed of microbial pigment reporting elements are more convenient than those composed of fluorescent protein reporting elements for color observation and is more suitable for the development of vision whole-cell biosensors. Common microbial pigments include carotenoid [27], melanin [28], linomycin [29], purple bacillus [30], indigo [31], and more. The performance of the microbial pigment reporting element depends on the condition of the substrate in the chassis cells. The number of genetic elements and the visual significance of the color change from substrate to product.

### 3.5. Gas Reporting Element

In recent years, whole-cell biosensors using gas as an output signal have emerged [32]. This is because the output signals of fluorescence and pigment reporting elements are optical signals; the application-specific detection system must be transparent, and cannot detect opaque samples, such as soil, grain, milk, etc. Whole-cell biosensors with fluorescence and pigment output signals can detect opaque samples only if the substances under test are separated, redissolved in a clear solution via pretreatment, or the whole-cell biosensors are separated and resuspended in a clear culture after incubation with the sample, but these above additional steps increase the detection complexity and reduce the detection accuracy. However, whole-cell biosensors consisting of gas reporting elements can spontaneously concentrate the output gas signals in the headspace of the detection system when detecting target substances, and are therefore suitable for both transparent and opaque samples. The *norB* gene (catalytic generation of nitrous oxide), *efe* gene (catalytic generation of ethylene) [33], *mht* gene (catalytic generation of ethylene) [34], and *dsr* gene (catalytic generation of methyl mercaptan) are common gas reporting elements. Liu et al. [33] developed a method utilizing gas as a reporting signal that could be used for the rapid on-site detection of mercury in soil. In this biosensor, the MerR protein acts as a sensing element that captures mercury ions and then binds the promoter of the *efe* gene to initiate the synthesis of the ethylene (C_2_H_4_)-forming enzyme that produced the gas. The results show that the mercury ion concentration can be converted into C_2_H_4_ gas signals, and the detection of C_2_H_4_ can effectively reflect whether the mercury pollution in soil exceeds the limit standard.

### 3.6. Magnetosome

In 1975, R.P. Blakemore, an American, first discovered a kind of bacteria whose movement direction can change with the polarity of the applied magnetic field and named it magnetotactic bacteria [35]. The magnetic nanoparticles in magnetotactic bacteria are magnetosomes, which are assembled into long chains and arranged along the long axis of the bacteria to guide the movement of the bacteria in the external magnetic field. In-depth studies of magnetosomes have revealed that if magnetosomes can be produced heterologously in engineered bacteria such as *E. coli*, most genes can be integrated into the *E. coli* genome for constitutive expression and a few key genes can be used as reporting elements for inducing expression [36]. When the target substance is present, the chassis cells can generate a complete magnetosome, releasing magnetic signals and reflecting the concentration of the target substance by detecting the magnetic strength. In recent years, because of special properties such as single magnetic domain, excellent biocompatibility, and surface modification, magnetosomes are attracting more and more attention from researchers in biology, medicine, paleomagnetism, geology, and environmental science [37]. In addition, magnetosomes can be applied in medical imaging [38], drug delivery [39], tumor hyperthermia [40], and wastewater treatment [41].

### 3.7. Ice Nucleation Protein

The phenomenon that water can remain liquid below 0 °C is called supercooling. Generally, to combat low-temperature stress, plants can maintain the water in their cells in a supercooled state at −6 to −7 °C, but there is a type of bacteria in nature that can induce the water in plant cells to freeze at −2 to −5 °C and cause frost. This kind of bacteria is called ice nucleation active bacteria. These bacteria effectively catalyze ice formation at much higher temperatures than most organic or inorganic substances. Since they are prevalent on the surfaces of frost-sensitive plants, they can cause icing, which results in frost injury. *Pseudomonas* and some species of *Owenella* are among the most commonly discovered ice nucleation active bacteria. Studies have shown that these ice nucleation active bacteria can produce a special protein with a specific repetitive sequence that can arrange water molecules into ice nucleation and then form regular, delicate, and tiny ice crystals. This protein is known as the ice nucleation protein. The ice nucleation gene is the gene encoding ice nucleation protein in ice nucleation active bacteria. When the ice nucleation gene is transferred into chassis cells, the ice nucleation protein can be measured to indicate the expression strength of ice nucleation gene expression. Therefore, the ice nucleation protein can be used as a reporting element. Loper et al. [42] monitored the utilization of iron by *Pseudomonas putida* (*P. putida*) using ice nucleation protein as the reporting element. The formation of ice nucleation protein is controlled by iron-regulated promoter (pvd). The determination of ice nucleation protein showed that siderophores produced by allogenic microorganisms enhanced the iron availability of *P. putida*. In addition, ice nucleation protein can be produced independently from ice nucleation protein detection due to its stable structure and long storage time. The ice nucleation protein reporting element can minimize the requirement on the physical properties of the sample, but the detection process is complicated and cannot achieve real-time dynamic monitoring.

### 3.8. Curli Protein

Curli, a functional amyloid protein found in *E. coli* and *Salmonella* biofilms, can self-assemble into nanoscale fibrin outside the cells. Studies have shown that Curli and similar functional amyloid proteins are key biofilm components that promote substrate adhesion, structural biofilm strengthening, and host cell invasion. Tay et al. [43] constructed a whole-cell biosensor to detect mercury ions by regulating Curli gene expression with MerR, a transcription factor specifically responsive to mercury ions. The optical density (OD) value of the supernatant after incubation with the biosensor cells showed a negative correlation with mercury ion concentration. Curli protein, as a special signal reporting element, could be used in the design and construction of visual whole-cell biosensors.

## 4. Genetic Circuits for Food Safety Detection Based on Synthetic Biology

**Figure 4:**
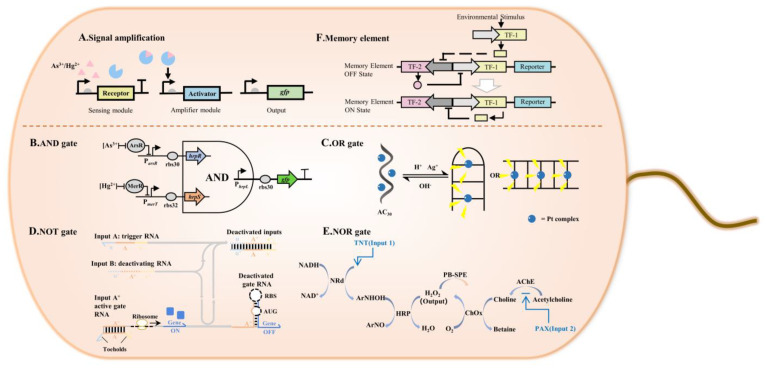
Gene circuits of synthetic biological whole-cell biosensors. (**A**) Adding a modular signal amplification module to a gene circuit can effectively reduce the detection limit and improve detection speed. (**B**) When all inputs are positive, the AND gate generates a report signal. (**C**) When any of the targets are positive, the OR gate generates a report signal. (**D**) When detection targets are negative, the NOT gate generates a report signal. (**E**) When all the targets are negative, the NOR gate generates a report signal. (**F**) Engineered bacteria with memory elements can sense, remember, and communicate their experiences, enabling them to detect and retain harmful substances in complex and dynamic situations, such as the mammalian gut.

The sensing and reporting elements are coupled through gene expression regulation to form a simple whole-cell biosensor gene circuit for direct single-target substance detection (Figure 4). However, sometimes a single-target substance is not sufficient to meet the needs of the detection. To realize the complex functions of the whole-cell biosensor system, such as signal amplification, multiple detections, and delayed reporting, it is necessary to build a complex whole-cell biosensor gene circuit with the help of other functional elements or modules composed of several functional elements to perform a certain task. Synthetic biological theory and technological developments are the primary impetuses for simple gene circuits’ evolution into complex ones. The following will introduce three complex gene circuits based on synthetic biology that can be used for food safety detection: signal amplification, logical calculation, and memory elements.

### 4.1. Signal Amplification

Synthetic biological whole-cell biosensors have great potential for detecting various food pollutants. However, whole-cell biosensors have a high detection limit and a long detection time, which is frequently insufficient to meet the requirements. Furthermore, adding a modular signal amplification module to the gene circuit is an effective solution to this problem (Figure 4A), as it can reduce the detection limit by two–three orders of magnitude and improve the detection speed. Signal amplification can be achieved using transcription factors, positive feedback loops, signal cascades, riboswitches, and a combination of several modalities.

#### 4.1.1. Amplified Signal Based on Transcription Factor Superposition

The P_laclq_ promoter is fused to the *rhlR* gene to amplify the response of the qsc promoter to acyl homoserine lactone (AHL), the efficient *cl(LVA)* suppressor gene is located downstream of the qsc promoter, and the expression of the fluorescent reporter is regulated by the λP_(R-O12)_ promoter. As a result of connecting these three gene circuits in series, Karig et al. [44] constructed a gene circuit for signal amplification. When exogenous C4HSL diffuses into the cell and binds to the rhlR receptor protein expressed by the RhlR gene, the C4HSL/RhlR complex activates the qsc promoter, resulting in Cl protein production, which regulates the expression level of the gene *eyfp*.

#### 4.1.2. Signal Amplification Based on a Positive Feedback Loop

Feedback can amplify the response to target substances and is a commonly utilized mechanism in regulating many gene circuits. Positive feedback has also been used to enhance the transcriptional activity of cells and tissue-specific promoters, thereby enhancing gene expression. Thus, positive feedback can be used in whole-cell biosensors. When the output signal of a certain target substance is weak, the positive feedback loop is able to amplify the output signal, making it easier for humans and instruments to detect. For example, Sayut et al. [45] used artificial positive feedback loops (PFLs) to enhance the reaction of weak promoters to achieve signal amplification. To construct the PFLs, the authors utilized the LuxR transcriptional activator and PluxI promoter isolated from the quorum-sensing system of the marine bacterium Vibrio fischeri. When a high concentration of 3-oxo-hexanoyl-homoserine lactone (OHHL) was added to the system, LuxR activated the PluxI promoter, which was upstream of LuxR and, thus, further promoted LuxR expression, resulting in positive feedback. The PFLs they constructed increased the sensitivity of the feedback loops, resulting in complete activation of the system at a concentration of 5 nM of OHHL, whereas wild-type PFLs were only fully activated at a concentration of 10 nM of OHHL. The application of artificial positive feedback loops in whole-cell biosensors can effectively complete the detection even when the concentration of a target substance is very low. In addition, Nistala et al. [46] designed an amplifier consisting of GFP and LuxR_Δ2-162_ arranged in series under the control of the PluxI promoter. The PluxI promoter activated transcription of LuxR_Δ2-162_, and LuxR_Δ2-162_ in turn activated the PluxI promoter, thus amplifying the signal. LuxR_Δ2-162_, therefore, functioned both as the input and positive feedback signal. The advantages are as follows: Firstly, the feedback mechanism only depends on a single gene and requires no additional regulation. Secondly, the circuit is also capable of amplifying any transcription signal. Thirdly, it can be designed as a component of more complex genetic circuits.

#### 4.1.3. Realize Signal Amplification through a Series of Signals

Riboswitch is one of the common sensing elements in whole-cell biosensors, which regulates transcription and translation through conformational changes. However, changing the binding site sequence of the riboswitch may destroy the function and limit its ability to regulate the output signals, so that signals can usually be detected by sensitive equipment in the laboratory. Therefore, the practicability of riboswitch in whole-cell biosensors is limited. However, biological circuits can solve the output limitation of riboswitch biosensors. The biological circuit connects the cell and the quorum signaling molecules together, allowing the use of biological components to “tune” the output response of the biosensor to produce an amplification component, which will be able to work together with the riboswitch to achieve the effect of signal amplification. For example, Goodson et al. [47] developed a signaling amplification system that uses a riboswitch to initiate signaling between the sensing strain and the reporter strain of *E. coli*. Quorum sensing signaling molecules biologically link sensing and reporting strains together. Compared with the fluorescence expression directly controlled by the riboswitch, the amplification system produces more fluorescence, improves the sensitivity of the detection system, and reduces the time required to detect a signal output.

Furthermore, RNA–protein hybrid input can also achieve signal amplification. Jang et al. [48] designed hybridized RNA protein inputs that combine riboswitches and orthogonal transcriptional repressors to respond to different signals. The mixed inputs include a riboswitch that acts as a ligand response RNA sensor and a transcription suppressor that acts as a protein regulatory factor. Transcript repressors are maximally expressed in the absence of ligands and inhibit the expression of reporter genes downstream of the homologous promoter operator. Conversely, in the presence of a suppressor, the homologous promoter operator is released, and the reporter is expressed in the ligand. The authors can easily adjust the signal strength by adjusting the promoter strength.

#### 4.1.4. Plasmid Copy Number Riboswitch-Based Signal Amplification

Two ligand-activated riboswitches that respond to the same ligand are introduced into a plasmid, one of which controls the expression of the target gene and the other controls plasmid replication. The addition of a ligand leads to the up-regulation of target genes and the increase in plasmid copy number, which results in the increase in fluorescence signal and the effect of signal amplification. Dwidar et al. [49] designed and constructed a new strategy for amplifying synthetic riboswitch output by simultaneously controlling the translation of the gene of interest and the plasmid copy number in bacteria. The results showed that the strategy could significantly improve the riboswitch in the dynamic range of gene expression and effectively achieve signal amplification. Additionally, since T7RNA polymerase (T7RNAP) genes are transcribed five times faster than *E. coli* genes, T7RNAP-based signal amplifiers have been used to increase signal output. Kim et al. [50] amplified the promoter/cell response to isoprene by designing a regulatory cascade coupling the PtbuA1 promoter to the activity of T7RNAP.

#### 4.1.5. The Signal Amplification System Is Constructed Using the Combination of Several Methods

In addition to the above methods, signal amplification can also be achieved by combining various methods, which has been reported. For example, Wan et al. [51] jointly developed a novel modular sensor signal amplification method through three methods and designed ultra-sensitive arsenic and mercury whole-cell biosensors, which increased the detection limit and output by 5000 and 750 times, respectively. The first method for increasing sensor sensitivity involves changing the intracellular receptor protein density in the sensor module or changing the strength of the constituent promoter (P_C_) in the sensor element. The strength of the P_C_ establishes receptor protein abundance (the weaker the promoter, the higher the sensitivity of the sensor and the larger the dynamic range). Moreover, the receptor protein inhibits ligand activity by repressing the cognate promoter (P_R_) from activating the transcription of an mRNA encoding a green fluorescent protein. The second method is to design a high-gain transcription amplifier in the computing module that amplifies the sensor signal from the P_R_, thereby increasing the detection range. The third approach is to connect multiple amplifiers in series to amplify the biosensor signals in order to increase the fluorescence level and reduce the time required for detection.

### 4.2. Logical Calculation (Logic Gate)

Multi-target detection is one of the development trends of whole-cell biosensors. When a whole-cell biosensor detects multiple targets, a parallel detection channel can be constructed, that is, each target corresponds to a signal reporting element. Signal integration can also be carried out, that is, the detection results of multiple targets can be integrated into a report signal through logical calculation. Modules that implement logical operations are called logic gates. Common logic gates include AND (Figure 4B), OR (Figure 4C), NOT (Figure 4D), and NOR gates (Figure 4E), etc. The operation rule of AND gate is to generate a report signal when all the targets are positive, and the operation rule of OR gate is to generate a report signal when any of the targets are positive. Additionally, the operation rule of NOT gate is to generate a report signal when detection targets are negative. Lastly, the operation rule of NOR gate is to generate a report signal when all the targets are negative.

#### 4.2.1. AND Gate

AND gate is one of the earliest logic gates. A basic AND gate require two inputs, and in more complex systems, two input gates can be combined to create AND gates with three or four inputs. When all inputs are positive, the AND gate generates a report signal. Wang et al. [52] designed whole-cell biosensors to detect pollution-related molecules and heavy metal ions using natural signal transduction mechanisms of chassis cells and bacteria. Using GFP as an indicator, they constructed a dual-input AND gated biosensor sensitive to arsenic (As^3+^) and mercury (Hg^2+^) ions. The hrpR and hrpS are driven by the arsenic and mercury sensors, respectively; the GFP is fused to the hrpL output promoter of the modular AND gate. The results showed that only when concentrations of arsenic and mercury were high enough could the two inputs of the genetic AND gate be activated. Genetic logic gates can be used as biofilters and amplification circuits to enhance the selectivity and sensitivity of whole-cell biosensors.

#### 4.2.2. OR Gate

The OR gate generates the report signal if any of the input targets are evaluated as positive. Silver (Ag) ions and silver nanoparticles (AgNPs) have found increasing applications in consumer products such as detergents, antibacterials, and wound dressings, raising concerns over their potential toxicity and bioaccumulation in our environment. In the meantime, acid rain is also a serious environmental problem due to its impact on the life forms of terrestrial and aquatic organisms. Ma et al. [53] described a light-emitting platinum (II) switched probe-based “OR” DNA logic gate that could simultaneously respond to AgNPs and H^+^ ions. The adenine and cytosine-rich oligonucleotide AC_30_ exists in solution as a single-stranded conformation in which platinum (II) complexes cannot be embedded in the H^+^ or Ag^+^ ions. The addition of H^+^ or Ag^+^ induced the conversion of AC_30_ from single-stranded to double-stranded, allowing the platinum (II) complex to bind to it and produce a strong phosphorescent response. Experiments confirmed that adding any input (pH ≤ 5 or AgNP ≥ 20 nM) will trigger the phosphorescence of the metal complex, resulting in a switched output. In addition, they used the system to detect nine different interfering metal ions (Ba^2+^, Na^+^, K^+^, Cr^3+^, Cd^2+^, Ni^2+^, Li^+^, Zn^2+^, and La^3+^) to demonstrate its selectivity. The results showed that there is only weak phosphorescence after the addition of interfering metal ions, and only the addition of Ag ions can significantly increase the phosphorescence intensity, indicating the high selectivity of the system for Ag ions. Due to the fact that low pH and the presence of Ag or AgNPs may be considered indicators of pollution, the logic gate can serve as a fast sensor for on-site estimation of water or soil quality without the need for expensive sensing equipment.

#### 4.2.3. NOT Gate

The operation rule of NOT gate refers to the generation of a report signal only when the detection target is negative. Green et al. [54] designed a logic gate model of a transcription factor “ribocomputing” system to evaluate complex logic in living cells. Ribocomputing systems are composed of de novo-designed parts and operate through predictable and designable base-pairing rules, allowing the effective in silico design of computing devices with prescribed configurations and functions in complex cellular environments. Based on base-pairing rules (DNA: A-T, C-G; RNA: A-U, C-G), the system designs computing devices with RNA molecules as input signals and proteins as output signals. The input RNAs can interact with themselves and the gate RNA. Input RNAs can interact cooperatively or competitively with one another to activate the gate RNA for AND logic or NOT logic, respectively. The NOT gate is accomplished by directly hybridizing the deactivated RNA with the trigger RNA to inhibit its action on the gate RNA. After binding to the gate RNA, the deactivated RNA can bind directly to free trigger RNA and use the extended single-stranded domain of the trigger RNA as a fulcrum to replace the trigger RNA. Successful operation of ribocomputing devices based on programmable RNA interactions suggests that systems employing the same design principles could be implemented in other host organisms or in extracellular settings.

#### 4.2.4. NOR Gate

A NOR gate consists of an OR gate and a NOT gate. The NOR gate operates only when both inputs are negative. For safety reasons, it is necessary to develop an easy-to-use, field-deployable kit that can quickly assess multiple chemical threats (i.e., explosives and nerve agents) and alert the operator when a hazard has been encountered. Chuang et al. [55] explored a novel biocatalytic cascade that stimulates NOR logic gates and can provide a simple and fast “yes/no” alert to detect the presence of explosive compounds and nerve agents. As model inputs, 2,4,6-trinitrotoluene (TNT) and paraoxon (PAX), as well as 2,4-dinitrotoluene (DNT) and methyl parathion (MPT), were used to investigate the detection of the versatility of enzyme logic gates for detecting a wide range of inputs. The logic gate employs a cascade of reactions catalyzed by four enzymes: nitroreductase (NRd), horseradish peroxidase (HRP), acetylcholinesterase (AChE), and choline oxidase (ChOx). These four enzymes are utilized as the backbone of the catalytic logic gate in order to process the TNT and PAX chemical inputs. Acetylcholine is catalytically coupled with AChE/ChOx to produce H_2_O_2_. Alternatively, in the presence of nitro-aromatic explosive substrates, the NRd/HRP biocatalytic cascade depletes H_2_O_2_ to some extent. In addition, organophosphate nerve agent inhibition of AChE can also reduce H_2_O_2_ levels. A Prussian blue (PB) modified screen-printed electrode was used to record the concentration of H_2_O_2_ as an output. PB, as a highly sensitive mediator to H_2_O_2_, can increase the efficiency of detection. When H_2_O_2_ levels drop below the selected decision threshold, a “hazardous” situation exists, indicating the presence of explosives and nerve agents consistent with the operation of the NOR gate. Compared with traditional detection methods, the method developed by the authors requires a shorter detection time.

### 4.3. Memory Element

Cells can use various biochemical mechanisms embedded in their regulatory networks to “remember” events. This helps detect and retain harmful substances in complex and dynamic situations such as the mammalian gut that can interact with the host to affect health, disease, and metabolism. Engineered bacteria with memory elements can sense, remember, and communicate their experiences while inhabiting the digestive tracts of mammals. (Figure 4F).

Based on the principle of synthetic biology, memory elements can be constructed artificially and applied in different fields such as health care, environmental monitoring, etc. There are two general ways of artificially constructing memory elements. Kotula et al. [56] used the most common mechanism in a switch, in which repressors inhibit the expression of another gene, a memory state corresponds to repressor dominance, and a feedback loop that maintains a memory state requires constant energy and materials for transcription and translation, similar to volatile memory in electronic circuits. They built a system in *E. coli* strains based on the bistable lambda cI/Cro switch. The circuit started in the cI state and switched to the Cro state induced by a “trigger” element in which a tetracycline-responsive promoter directed transcription of a Cro gene. When *Escherichia coli* bearing the memory system were administered to mice treated with anhydrotetracycline, the recovered bacteria had all switched to the Cro state, whereas those administered to untreated mice remained in the cI state. This demonstrated the successful construction of memory elements. The second method uses recombinant enzymes to bind two recognition sites and transform the DNA in the middle, preserving the state corresponding to these two directions even after the cell dies. For example, Yang et al. [57] used the irreversible large serine phage (LSTP) integrase, which mediated the integration and excision of phages between their homologous recognition sites attB (bacteria) and attP (phage), to cut, rotate, and reconnect DNA. Placing these sites in opposite directions reverses the regions between sites, thereby building a storage array capable of recording 211 (2048) combinations of states (1.375 bytes of information).

## 5. Food Safety Testing System and Commercial Presentation Form Based on Synthetic Biology

Gene circuit realization in synthetic biology requires ATP for energy, ribonucleotides, and amino acids for raw materials, and RNA polymerase and ribosome for catalysis, indicating that it must occur in an intracellular or similar environment with similar characteristics. Microorganisms, animal, and plant cells can be used as the chassis cells of whole-cell biosensors. However, due to their ease of cultivation, rapid reproduction, and relatively simple metabolism, microbial cells are the most widely used chassis cells of synthetic biological whole-cell biosensors at present.

In recent years, cell-free protein expression systems have inspired synthetic biological cell-free biosensor development, which uses cell fragmentation fluid, cell extraction fluid, and artificially formulated cell simulators as environments for synthetic biological gene circuits. Many biosensors, such as aptamer sensors and antigen-antibody response sensors, are also biological elements that function in a cell-free environment. The term cell-free biosensor does not accurately reflect the distinction between different types of biosensors. Therefore, biosensors that mimic the function of cellular sensors but do not operate in intact cells and are based on the transcription-translation of gene circuits in non-holonomic cell environments are cell-like biosensors. Therefore, we suggest that such biosensors based on the transcription-translation of gene circuits in non-holonomic cell environments be named cell-like biosensors. The difference between cell-like biosensors and whole-cell biosensors lies in that whole-cell biosensors are built in a complete cellular environment, while cell-like biosensors mimic the working principle of whole-cell biosensors in non-holonomic cells.

### 5.1. Microbial Cell Sensing System

Choosing the appropriate chassis cells is one of the key points for successfully constructing microbial whole-cell biosensors. Most microbial whole-cell biosensors currently use engineered *E. coli* as chassis cells. Engineered *E. coli* has a clear genetic background, an abundance of plasmid tools and gene editing tools, rapid growth, low culture costs, and relative safety. The simplified *E. coli* genome can reduce background gene interference and potential safety risks and improve whole-cell biosensor efficiency [58]. The living environment of *E. coli* is relatively wide, and the detection function can be completed under normal circumstances. However, in some special detection environments or detection environments with stringent food safety requirements, *E. coli* is not the best choice. The selection of the original strain in the sample as the chassis cell of the biosensor may be more conducive to the stability and accuracy of the detection results. (1) *E. coli* native soil strains, including *Agrobacterium* [59], *Shivanella* [34], *Pseudomonas* [60], *Enterobacter* [61], and *Rolstone’s* bacteria [62], can be used to detect contaminants in soil samples. For example, in order to investigate quorum sensing in rhizosphere soil, DeAngelis et al. [59] constructed an *Agrobacterium tumefaciens* (pAHL-Ice) whole-cell biosensor using *Agrobacterium tumefaciens* as chassis cells to detect N-acyl homoserine lactones (AHLs). (2) Fermentation strains can be selected for in situ real-time detection of fermented products. For example, in shikimic acid fermentation, *Corynebacterium glutamicum* can be used to construct whole-cell biosensors to detect shikimic acid [63]. During Menaquinone fermentation, *Bacillus subtilis* was used to construct whole-cell biosensors to detect menaquinone-7 [64], while *Saccharomyces cerevisiae* was used to construct whole-cell biosensors to detect naringenin during the naringenin fermentation [65].

### 5.2. Cell-Like Sensing System

Cell-like biosensors are biosensors that mimic the working principle of whole-cell biosensors but do not work in intact cells. The establishment of a cell-like sensing system mainly involves the process of cell fragmentation to obtain cell extract. Common cell fragmentation methods include ice bath ultrasonic, ball mill, liquid nitrogen, and electric shock fragmentation. Adding a synthetic plasmid containing a synthetic biological gene circuit and any other required substances to the broken extract creates a sensing system that functions similarly to a cell. Compared with the microbial whole-cell biosensing system, the cell-like sensing system breaches the cell boundary, surpassing the restrictions of the cell wall and cell membrane, which provides the following advantages: firstly, it simplifies the process of the target substance through the cell wall and membrane, improves the probability of the sensor capturing the target, improves the detection efficiency, and reduces the detection time. Kathryn et al. [66] established three kinds of cell-like biosensors for detecting arsenic, cadmium, and mercury, two of which reached the WHO target detection levels and all of which achieved the US MEG levels. In addition, the detection time was shorter than other detection methods. Secondly, the ionic environment regulation of the detection system was easier to implement. Some biological elements or methods that are difficult to be applied in cells, such as aptamers screened in vitro, nucleic acid elements that are easily degraded, and cross methods based on Polymerase Chain Reaction (PCR) and CRISPR-Cas9 [67], etc., are easier to apply in cell-like sensing systems. Thirdly, cell-like biosensors are less affected and more toxicity-resistant to some toxic target substances with genotoxic and oxidative stress toxicities. However, whole-cell system destruction also leads to the loss of some traditional advantages of whole-cell biosensors, such as the rising production costs of cell-like biosensors and poor resistance to detect environmental changes. In summary, both whole-cell and cell-like biosensors have advantages, and it may be possible to form two distinct development lines in the future.

### 5.3. Commercial Presentation Form

**Figure 5:**
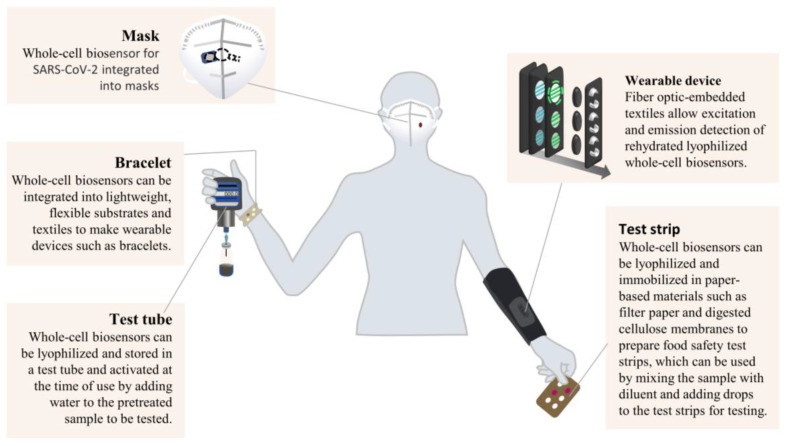
Commercial presentation forms of whole-cell biosensors based on synthetic biology.

Whole-cell or cell-like biosensors based on synthetic biology have the advantages of low cost, strong anti-interference ability, and simple operation, so they are theoretically suitable for application in food safety on-site detection. Whole-cell biosensors are developed in the laboratory. To enable the application of whole-cell biosensors for commercial on-site detection, it is necessary to present them on a platform. Test tubes, masks, bracelets, wearable devices, and test strips are portable platforms that can be docked with synthetic biological whole-cell biosensors (Figure 5). For example, as cells are cultured in test tubes, most whole-cell biosensors are performed in test tubes during the development phase. In commercial applications, whole-cell or cell-like biosensors can be freeze-dried and stored in test tubes, which are activated by adding water and pre-treated samples for detection. Whole-cell biosensors and cell-like sensors can also be freeze-dried and immobilized in filter paper, nitrocellulose membrane, and other paper-based materials to prepare food safety test strips. Samples and diluent are mixed and then dripped onto test strips for detection [68]. The test strips are cheaper, more portable, and safer than test tubes. Recently, Nguyen et al. [69] reported the integration of whole-cell and cell-like biosensors into soft textiles, which they used to make wearable devices such as hand rings and clothing. Integrating synthetic biological whole-cell biosensors into wearable devices could improve their non-invasive monitoring capabilities of physiological states, disease states, and pathogen and toxin exposure.

## 6. Summary and Outlook

The elements and functional modules supplied by synthetic biology provide more choices for food safety detection in special application scenarios. For example, when conducting on-site testing on opaque samples, the utilization of optical reporting elements will be greatly limited, and gas reporting elements and magnetic reporting elements will be better choices. Additionally, when conducting high-throughput detection or multi-targets detection, the logic gate element can detect signals of different targets and give a comprehensive judgment on whether there are pollutants in food samples. Furthermore, when delayed detection is required, such as using biosensor cells to detect pollutants in the gut, the biosensor cells need to be isolated from the stool to obtain the results. In this case, memory elements are necessary.

### 6.1. Challenges for Synthetic Biological Whole-Cell Biosensors

Despite the rapid development of synthetic biological whole-cell biosensors, their commercial application still faces three major technical obstacles. Above all, compared with other rapid food safety detection methods, whole-cell biosensors based on synthetic biology are notably slower. Whole-cell biosensors based on bacteria require 20 min to several hours from the addition of samples to obtain results, while whole-cell biosensors based on eukaryotic microorganisms such as yeast require hours to several days. Synthetic biological detection of whole-cell biosensors usually requires multiple links, such as RNA transcription, protein translation, and product accumulation, which slows the detection rate. The detection speed of cell-like sensors has improved compared with whole-cell biosensors, but it is still difficult to match the detection speed of quantum dot sensors and other technologies. This drawback requires synthetic biological development and application of functional nucleic acid elements, such as aptamer sensing elements, RNA signal amplification elements, and fluorescent aptamer reporting elements. Afterward, since synthetic biological cell biosensors are constructed based on genetically modified microorganisms, post-detection processing is very crucial in commercial applications, and inadvertent handling may lead to biological risks. In R&D laboratories, synthetic biological whole-cell biosensors can usually be treated by high temperature and high pressure sterilization after detection, and the cells and their gene circuit fragments will not diffuse into the environment. However, in commercial applications, it is difficult to destroy gene circuit fragments of cells, and there is a risk of transfer and integration into other microbial genomes, resulting in biosafety risks. Combining synthetic biological whole-cell biosensors with nano-material technology is necessary for technological breakthroughs in this field. For example, the cell and its nucleic acid cannot escape from the nano-cage after the detection, but degrade slowly in it [70]. Finally, protecting the intellectual property of synthetic biological whole-cell biosensors is also a crucial concern. The advantage of synthetic biological whole-cell biosensors is that they are easy to produce. Meanwhile, after receiving commercial synthetic biological whole-cell biosensor products, purchasers readily isolate cells from the product and expand the culture without purchasing a similar product again. This problem can be solved by constructing chassis cells that rely on specific nutrient factors so that purchasers who are not aware of the nutrient factors cannot cultivate the cells themselves.

### 6.2. Development Direction of Synthetic Biological Whole-Cell Biosensors

Future developments in synthetic biological whole-cell biosensors will focus on target diversification, function expansion, and intelligent design. First of all, target diversification refers to a whole-cell biosensor that can detect multiple target substances, thereby significantly improving the detection efficiency. The implementation of simultaneous detection of multiple targets relies on the integration of multiple target signals through logic computing gene circuits, and it is necessary to solve the problem of signal interferences between each target. Hao et al. [71] developed a pH-resolved colorimetric biosensor that can simultaneously detect four targets: ochratoxin A (OTA), aflatoxin B1 (AFB1), fumonisin B1 (FB1), and microcystin-LR (MC-LR). By adjusting the pH of the solution, the concentrations of the four targets can be obtained in turn. Secondly, function expansion refers to the expansion of the functions of synthetic biological whole-cell biosensors besides detection functions, such as the expression of protease, lipase, phytase, etc., to complete the digestion function of food organics, and the expression of enzymes to complete the target transformation function. These functional extensions simplify the sample pretreatment process required for synthetic biological whole-cell biosensors. Finally, intelligent design refers to the intelligent and customized design of gene circuits of synthetic biological whole-cell biosensors through functional gene element databases, standardized element interfaces, and automated design tools. Berepiki et al. [72] used the design of experiments (DoE) method to effectively map gene expression levels and provide performance-enhancing biosensors. The DoE method can increase the maximum signal output, improve the dynamic range, expand the sensing range, improve the sensitivity, and systematically modify the dose–response behavior of biosensors to provide a design of biosensors with both digital and analog dose–response behavior.

## Figures and Tables

**Table 1:** Comparison of whole-cell biosensors’ reporting elements.

Reporting Element Type	Classification	Output Signal	Substrate Requirement	Testing System Requirement	Signal Maintenance Time	Signal Visualization
Luciferase	Bacterial luciferase	fluorescence signal	endogenous/exogenous	transparent	real-time signal	no
	Firefly luciferase					
	Aequorin					
Fluorescent protein	Dark blue	fluorescence signal	no substrate required	transparent	real-time signal	strong signal visual
	Blue					
	Cyan					
	Green					
	Yellow					
	Orange					
	Red					
	Dark red					
	Near infrared					
Fluorescent aptamer	Malachite green aptamer	fluorescence signal	exogenous	transparent	real-time signal	no
	Spinach aptamer					
	Mango aptamer					
	Broccoli aptamer					
Microbial pigment reporting element	Carotenoid	pigment signal	endogenous/exogenous	transparent	cumulative signal	visual
	Violacein					
	Melanin					
	Prodiginine					
	Indigoidine					
	Betaxanthin					
Gas reporting element	N_2_O	gas signal	endogenous/exogenous	no gas adsorption	real-time signal	no
	CH_3_Cl, CH_3_Br, CH_3_I					
	CH_3_SH					
	C_2_H_4_					
	CH_3_CHO					
Magnetic reporting element	Magnetosome	magnetic signal	endogenous	no	real-time signal	no
	MagA					
	Metallothionein and Phytochelatin synthase					
	Ferritin					
Other reporting element	Ice nucleation protein	ice nucleation protein	no substrate required	no	cumulative signal	no
	Curli protein	bacteriophage sedimentation	no substrate required	transparent, liquid	cumulative signal	visual

## Data Availability

No new data were created or analyzed in this study. Data sharing is not applicable to this article.
